# Decision-Making Skills in Youth Basketball Players: Diagnostic and External Validation of a Video-Based Assessment

**DOI:** 10.3390/ijerph18052331

**Published:** 2021-02-27

**Authors:** David Rösch, Florian Schultz, Oliver Höner

**Affiliations:** Institute of Sports Science, Eberhard Karls University of Tübingen, 72074 Tübingen, Germany; florian.schultz@uni-tuebingen.de (F.S.); oliver.hoener@uni-tuebingen.de (O.H.)

**Keywords:** talent identification, team sports, diagnostic instrument, perceptual-cognitive skills, game-related statistics

## Abstract

Decision-making is a central skill of basketball players intending to excel individually and contribute to their teams’ success. The assessment of such skills is particularly challenging in complex team sports. To address this challenge, this study aimed to conceptualize a reliable and valid video-based decision-making assessment in youth basketball. The study sample comprised youth basketball players of the German U16 national team (*n* = 17; *M_Age_* = 16.01 ± 0.25 years) and students of a sports class (*n* = 17; *M_Age_* = 15.73 ± 0.35 years). Diagnostic validity was tested by determination of the performance levels according to response accuracy as well as response time in the assessment. External validity was examined by investigation of the correlation between the diagnostic results of the elite athletes and their real game performance data associated with passing skills. Logistic regression analysis revealed that the diagnostic results discriminate between performance levels (χ^2^(2) = 20.39, *p* < 0.001, Nagelkerke’s *R^2^* = 0.60). Multiple regression analysis demonstrated a positive relationship between the diagnostic results and assists (*F*(2,10) = 4.82, *p* < 0.05; *R^2^* = 0.49) as well as turnovers per game (*F*(2,10) = 5.23, *p* < 0.05; *R^2^* = 0.51). However, no relationship was detected regarding the assist-turnover ratio. Further, response time discriminated within the elite athletes’ performance data but not between performance levels while for response accuracy the opposite is the case. The results confirm the diagnostic and external validity of the assessment and indicate its applicability to investigate decision-making skills in youth basketball.

## 1. Introduction

The performance of a basketball player and the success of a team is particularly based on passing skills because promising shooting options are created through appropriate passing decisions and cooperation [1]. In this context, the point guard has a distinguished role as he is typically organizing the teams’ tactical patterns. One of his main tasks is to pass the ball to his teammates in promising positions where they can exhibit their unique skillsets. Therefore, point guards make significantly more passes than players in other positions with youth basketball teams averaging over 256 passes each game [2]. However, the evolution of the game through rule changes led to the adjustment of the requirements on all playing positions [3] and altered the way the game is played today [4]. That is, for example, that in the modern game of basketball, players aside from the point guard (e.g., power forward or center) are also required to take responsibility for distributing the ball. This highlights the importance of passing skills, which remain as pivotal for performance in basketball.

In general, sports performance is moderated by multiple interdependent variables including physiological, biomechanical, and psychological factors [5]. In the past years, extended research concerning physical factors was conducted [6] while the role of other performance factors such as psychological factors attracted less attention [7,8]. To specify, these factors incorporate psychomotor, cognitive, and personality-related factors. Decision-making is one cognitive factor that is considered a central contributor to performance in team sports in connection to other cognitive factors like perceptual-cognitive skills or executive functions [9]. The assessment of performance and recognition of potential talent is a major challenge [10] particularly in complex team sports such as basketball [11]. Therefore, talent identification traditionally relies on the knowledge and perceptions of expert coaches [12]. Scientific evidence, especially regarding cognitive factors, could support coaches in this process of talent identification [13]. Thus, the current study focuses on decision-making which generally refers to the process of making a choice from a set of options, with the consequences of that choice being crucial [14]. With respect to team sports such as basketball, decision-making is defined as the ability to perceive essential information from the playing environment, correctly interpret this information, and then select the appropriate response [15]. This is exemplified by a passing decision in basketball: A player in possession of the ball initially has to perceive where his teammates and their respective defenders are located on the court. Subsequently, he has to recognize the open teammate before finally executing a pass.

In sports-related research on cognitive performance factors, two approaches are predominant: The *cognitive component skills approach* [16] is employed to examine fundamental cognitive factors such as the athlete’s executive functions (i.e., cognitive flexibility, working memory, inhibition). Therefore, tests are conducted that are unspecific to the respective sporting domain (e.g., Multiple Object Tracking) [17]. Research with a focus on this approach found superior cognitive functions in high-performance level athletes compared to low-performance level athletes [18]. These functions are assumed to contribute to the successful accomplishment of the complex demands in competitive sports [19]. However, it is still unclear whether this superiority in cognitive functions characterizes sport-specific expertise or results from extensive practice. In contrast, the *expert performance approach* [20] is applied to investigate the athletes’ cognitive performance factors in a sport-specific context. For this purpose, more ecologically valid tasks are conducted to examine the athletes’ behavior in their respective domains. Using this framework, it has been established that elite athletes possess superior perceptual-cognitive skills when compared to intermediate and non-elite performers [21,22,23]. The application of this approach in a basketball-specific context revealed expertise effects in anticipation [24,25,26,27], pattern recall [28,29,30,31,32], and visual search strategies [33,34]. Advantages are also reported when focusing on decision-making skills: Spittle et al. [35] found that participants who currently played basketball or played at a higher level of competition made more correct decisions in a video-based test than those currently not playing or doing so on a lower level of competition. These findings are supported by Gorman, Abernethy, and Farrow [29] who conducted a decision-making task with static and moving videos and demonstrated higher response accuracy in experts compared to novice basketball players. Besides higher response accuracy, Ryu, Abernethy, Mann, Poolton, and Gorman [34] found faster response times for skilled compared to less skilled basketball players in a video-based task. Current research in this field also provided evidence for the benefits of advanced technology (i.e., virtual reality environments) to improve decision-making skills in youth and senior basketball players [36,37].

Decision-making skills are commonly assessed in laboratory settings by applying either the reaction-time paradigm, temporal or spatial occlusion approaches [38]. During such investigations, the athlete is watching video scenes of relevant game scenarios and must respond either as quickly as possible or as precisely as possible after the scene was terminated at a certain time point. A central criticism of laboratory studies with these approaches is the lack of representation of the sport-specific reality [21,39]. This is also apparent in previous basketball-related research with a focus on decision-making skills [29,34,35] that utilized video scenes filmed from a third-person perspective which does not replicate the on-court reality. In addition, the viewing point of such stimuli has been shown to influence decision-making skills [40]. However, it has been noted that laboratory studies allow greater experimental control than in-game investigations [23], especially because of the presentation of standardized in-game scenarios [41]. Thus, the application of these approaches appears reasonable while addressing the outlined criticism. To increase the diagnostic validity of a video-based assessment for decision-making, the employment of video scenes from the first-person perspective in contrast to previously used stimuli is feasible. Furthermore, it is inevitable to address external validity to provide a representative task and enable the transfer of the diagnostic results to different populations or settings [42]. In this context, the application of sports data (i.e., data from sports tournaments) is convenient since such data reflect the behavior of experienced professionals and therefore retain sufficient external validity [43]. Within the domain of basketball, comprehensive datasets are available, because, in every official basketball game on international youth and senior level, performance data are recorded. These statistics provide detailed information on the performance of each player and the competing teams such as points scored or shooting percentages. Regarding decision-making in passing situations, it is interesting to focus on the variables of assists and turnovers. These parameters are appropriate to measure the ball control of a player [44] as well as the coordination of a team [45] and are therefore relevant in this context. Besides, these measures have shown predictive validity for the success of youth [46] and senior [47,48] basketball teams. However, the basketball-related research concerning the linkage between cognitive factors and performance data is sparse. Only two studies examined the relationship between the performance of adult basketball players in a multiple object tracking task and their performance data [44,49]. Both studies were able to demonstrate a relationship between fundamental cognitive skills and performance data associated with passing skills. However, to the best of our knowledge, no study used performance data for external validation of a decision-making assessment in youth basketball.

Although former research established the validity of diagnostic instruments for the video-based assessment of decision-making skills in basketball, such tests are rarely used in the practical context. To support coaches evaluating talented youth players such a tool is eligible in addition to already established testing instruments (e.g., physical fitness tests) [50]. Therefore, this study particularly focuses on the conceptualization of an assessment for decision-making skills in passing situations incorporating video stimuli from a first-person perspective and the evaluation of the assessments’ diagnostic and external validity by using basketball players of a youth national team. This was pursued through two objectives: First, we determined the relationship between the diagnostic results and the performance level to evaluate the diagnostic validity of the assessment. Second, we investigated the correlation among the diagnostic results of the youth national team players and their performance data to examine the assessments’ external validity. The following hypotheses were tested:

**Hypothesis** **1** **(H1).** *The diagnostic results of all youth athletes (i.e., response accuracy and response time) discriminate the performance levels (i.e., elite or non-elite).*

**Hypothesis** **2** **(H2).** *The diagnostic results of the youth national team players (i.e., response accuracy and response time) correlate with their performance data associated with passing skills (i.e., assists, turnovers, assist-turnover ratio).*

## 2. Methods

### 2.1. Sample and Design

The sample of this quasi-experimental study comprised two subsets of male youth athletes: Youth basketball players of the German U16 national team (*n* = 17; *M_Age_* = 16.01 ± 0.25 years; positions: six guards, six forwards, and five centers) and students of a sports class (*n* = 17; *M_Age_* = 15.73 ± 0.35 years). The participants were selected according to the following criteria: Youth athletes (a) of the same age group (b) with high-level or largely without competitive experience in basketball were included while (c) experience in other sporting domains was required in the students. The youth basketball players competed in the highest national and international basketball competitions in this age group. In contrast, only two of the students had competitive experience in basketball. The students attended the 10th grade of a secondary school where physical education is a major subject and athletes from different sporting fields are supported to combine education with competitive sports. In line with most research, the youth athletes were categorized referring to the basketball-related performance level [51]. Accordingly, the youth basketball players were defined as *elite*, while the students were labeled as *non-elite*, both relative to their peer group. Participants and their parents provided informed consent for the collection and scientific use of the data. In addition, the study was approved by the university’s ethics department.

### 2.2. Stimulus Development

**Figure 1:**
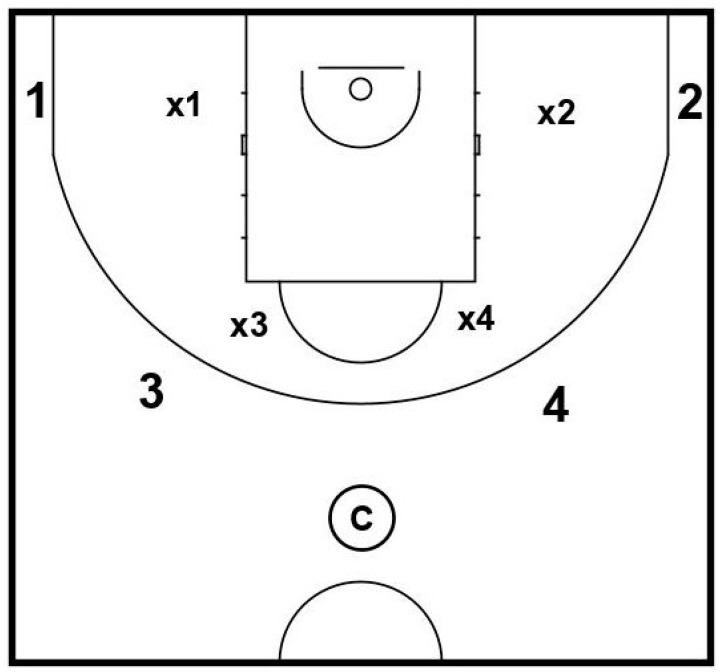
Initial setup of the players in the video scenes of the decision-making assessment. **Note.** Camera (C); Offensive players (1, 2, 3, 4); Defensive players (x1, x2, x3, x4). Numbers are not related to playing positions.

The scenes used in the assessment were filmed with a high-definition camera (Panasonic HDC-SD900, Panasonic Corporation, Kadoma, Osaka, Japan) including wide-angle-lens (Panasonic VW-W4607, Panasonic Corporation, Kadoma, Osaka, Japan) and showed a youth team from the highest youth league in Germany (Nachwuchs Basketball Bundesliga, NBBL, U19). The scenes display 4 vs. 4 game situations which were recorded in one-half of the court (see Figure 1).

**Figure 2:**
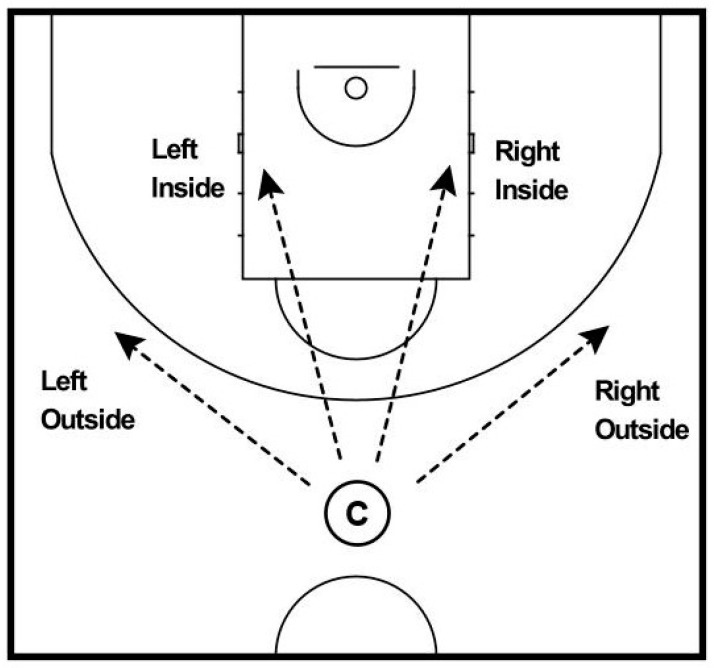
Passing options in the decision-making assessment. **Note.** Camera (C).

The camera represented the point guard as an additional player in ball possession. No defender was displayed in front of the camera as the participants would not have been able to overcome vision impairment by changing their point of view during the assessment. Each scene started with the same tactical setup but varied regarding the movement of the players and the best passing option. There were four proper passing options: right outside or inside, left outside or inside (see Figure 2).

Three coaches which hold the highest German basketball coaching license rated the video footage before the selection of the scenes. The coaches were asked to evaluate the scenes regarding representation and to determine the best passing options. If one of the coaches considered a scene not suitable or if the coaches disagreed upon the best passing option, the scene was excluded. Overall, the coaches evaluated 43 scenes of which 22 scenes were selected for the study (1 example scene, 3 practice scenes, 18 assessment scenes). The example and practice scenes display each passing option once. As a result of the expert rating, the proper passing options within the assessment scenes are not equally distributed (61.11% right side: 3 scenes outside, 8 scenes inside; 38.89% left side: 3 scenes outside, 4 scenes inside). The reliability of the assessment scenes was evaluated using the split-half method (Spearman-Brown corrected). The analysis revealed satisfactory results for response accuracy (*r* = 0.92) and response time (*r* = 0.84) indicating internal consistency of the decision-making assessment.

### 2.3. Procedures

**Figure 3:**
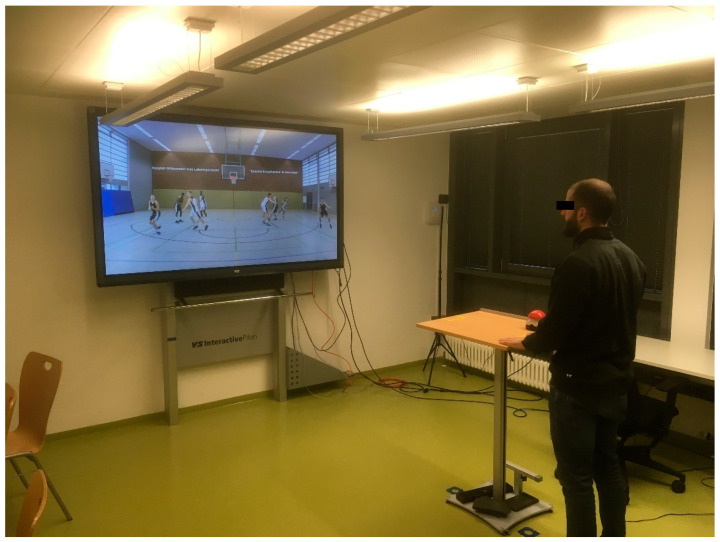
Experimental setting in the cognitive laboratory.

The scenes were presented to the elite athletes on a wall via a projector (ViewSonic PJD7820HD, ViewSonic Corporation, Walnut, CA, USA) and to the non-elite athletes on a smartboard (VS Interactive Intelligent Panel VSH84EA, VS Vereinigte Spezialmöbelfabriken GmbH & Co. KG, Tauberbischofsheim, BW, Germany). Different equipment was used for stimulus presentation since the elite athletes were tested during training camp while the non-elite athletes were tested in the university’s cognitive laboratory (see Figure 3).

Stimulus presentations were similar regarding quality (Full HD, 1920 × 1080 pixels) and format (16:9) but different regarding screen size (Projector: 257.0 × 144.6 cm; Smartboard: 186.0 × 104.6 cm). Previous research indicates that screen size does not influence decision-making accuracy when comparing the presentation on large and small screens [35]. In both presentation modes of the current study, the screen had sufficient size and thus no influence of screen size is presumed.

The study was organized in two sessions which were concluded in a total of approximately 30 minutes. In the first session, a visual response time task was conducted to control for general response time as a confounding variable. No significant differences (*t*(32) = −1.50, *p* = 0.14) between elite (*M* = 546.50 ± 53.84 ms) and non-elite (*M* = 577.97 ± 67.86 ms) athletes were detected computing a t-test for independent variables. Consequently, general response time was not further considered as a covariate. In the second session, participants received standardized visual and audible instructions for the setting. This was followed by one example and three practice trials to accustom the participants to the environment. The main investigation comprised 36 trials in two randomized blocks (18 different scenes presented twice). All participants were required to take their initial position standing in front of a desk with a mounted buzzer (Eaton FAK-R/KC11/I, Eaton Industries GmbH, Bonn, NW, Germany) and position their hand in a marked area on the desk. To assess the participants’ decision-making skills, the reaction-time paradigm [38] was applied. For every scene, the participants were asked to decide as fast as possible where to pass the ball. At the moment of their decision, participants were asked to press the buzzer first and give their answer orally within two seconds subsequently. A scene was terminated immediately with the participants pressing the buzzer. If the buzzer was not pressed before the offensive players finished their tactical pattern the screen turned black and the participants’ decision was considered incorrect.

### 2.4. Measures

#### 2.4.1. Decision-Making Assessment

Two dependent measures were captured in the decision-making assessment: response accuracy and response time. The participants’ oral responses were registered manually by the investigator. Response accuracy was measured as the percentage of correct decisions in all scenes. Response time was registered computer-assisted in milliseconds (ms) when the participant pressed the buzzer. Due to the different lengths of the scenes, response times were z-standardized in reference to the elite athletes. For this purpose, the athletes’ response time in a scene was subtracted from the mean response time of the elite athletes and divided by the standard deviation. Consistently, response time is negatively coded which means that lower values represent higher performance. For every scene, only response times were included which did not differ more than three standard deviations from the mean response time of the elite athletes [52].

#### 2.4.2. Performance Data

The elite athletes’ performance data from 15 international games were analyzed. Only those players were evaluated who competed in at least 20% of the games (*n* = 13; *M_Age_* = 16.00 ± 0.28 years; positions: four guards, five forwards, and four centers). Seven games took place at the 2019 FIBA U16 European Championship while a total of eight games were played in preparation for this tournament. In every official basketball game of the International Basketball Federation (FIBA), performance data are recorded software supported by trained staff. The collected data of each game is provided publicly in official FIBA box scores (see Supplementary Materials, Table S1). The datasets contain game-related information about several relevant indicators such as minutes played, points scored, or shooting percentages. Assists and turnovers were selected from the data. The assist-turnover ratio was computed as the quotient of those two variables. Assists are passes that lead to a direct score while turnovers are technical or tactical mistakes (e.g., misdirected passes) that lead to a change in ball possession. The assist-turnover ratio indicates that if a players’ ratio is higher than 1, he makes more passes that lead to a direct score than losing possession of the ball. If a player’s ratio is lower than 1, the opposite is the case.

### 2.5. Statistical Analysis

All data were analyzed using IBM SPSS Statistics Version 26 (IBM Corporation, Armonk, NY, USA). Multivariate logistic regression analysis was performed to investigate the diagnostic validity of the assessment. The performance level (non-elite = 0, elite = 1) was selected as the binary criterion variable while the diagnostic results (i.e., response accuracy and response time) were included as predictor variables. The overall model fit was analyzed with the likelihood ratio chi-squared test and Nagelkerke’s *R*^2^. As the contextual interpretation of Nagelkerke’s *R*^2^ is controversial due to the lack of generally accepted guidelines [53], additionally the odds ratios (ORs) *e^b^* and their 95% confidence intervals (CIs) were computed referring to the elite performance level. Furthermore, the external validity of the assessment was examined computing multiple regression analysis. This analysis also included the diagnostic results as predictor variables while the performance data (i.e., assists per game, turnovers per game, and assist-turnover ratio) were used as external criterion variables.

## 3. Results

Table 1 displays the descriptive statistics for the predictor variables of the logistic regression analysis.

Regarding diagnostic validity, this analysis confirmed a significantly better model fit compared to the null model (χ^2^(2) = 20.39, *p* < 0.001, Nagelkerke’s *R*^2^ = 0.60) and revealed that the diagnostic results discriminate the performance levels (H1). The results of the logistic regression analysis are presented in Table 2.

Within the model, the response accuracy explained the discrimination of performance levels significantly (*p* < 0.01). In contrast, the response time did not further contribute to the explanation of the model (*p* = 0.99). The ORs for the multivariate model demonstrated that a 10% higher response accuracy tripled the odds to be affiliated with the elite performance level ((*e^b^*)^10^ = 3.39).

Table 3 displays the descriptive statistics for the predictor and external criterion variables of multiple regression analysis.

With respect to external validity, this analysis demonstrated that the models addressing assists per game (Model 1: *F*(2,10) = 4.82, *p* < 0.05; *R^2^* = 0.49) and turnovers per game (Model 2: *F*(2,10) = 5.23, *p* < 0.05; *R^2^* = 0.51) as criterion variables showed significance (H2). Only the model addressing the assist-turnover ratio (Model 3: *F*(2,10) = 2.01, *p* = 0.19; *R^2^* = 0.29) as criterion variable failed significance. The results of the multiple regression analysis are displayed in Table 4.

**Figure 4:**
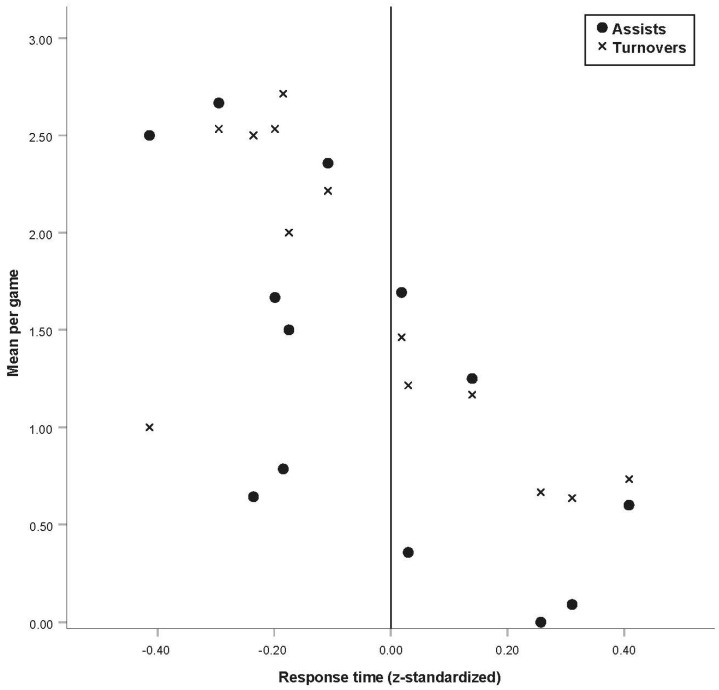
Association between the response time and the performance data. **Note.** For each player’s response time in the decision-making assessment, his mean assists (circle) and turnovers (cross) per game are represented.

The response time significantly contributed to the explanation of assists and turnovers per game (each *p* < 0.05). However, the response accuracy did not further contribute to the explanation of these models (Model 1: *p* = 0.79; Model 2: *p* = 0.80). While response time yielded an expected negative contribution to the explanation of assists per game this predictor unexpectedly also contributed in this direction to the explanation of turnovers per game. This is illustrated in Figure 4 showing an overlay plot of mean assists and turnovers per game for each player’s mean response time. The plot highlights that players who react faster in the assessment have both more assists and turnovers per game.

## 4. Discussion

This study aimed to conceptualize an assessment for decision-making skills in passing situations in basketball incorporating video stimuli from a first-person perspective and to evaluate the assessments’ diagnostic and external validity. Therefore, elite and non-elite youth athletes completed the assessment to determine the relationship between the diagnostic results and the performance level. In addition, the correlation between the diagnostic results of the elite youth athletes and their real game performance data associated with passing skills was examined.

As a prerequisite, the internal consistency of the assessment was confirmed in this study. The reporting of reliability indicators is essential to provide high precision of diagnostic instruments and enable the replication of results as well as the comparison between assessments [54]. Former research developing video-based decision-making instruments widely did not report indicators concerning reliability [55] which limits the comparison of the reliability indicators.

Concerning the first objective, the results confirmed the diagnostic validity of the assessment. The diagnostic results discriminated significantly between the two performance levels (H1). Within the model, response accuracy was the main contributor to the explanation of the performance levels. This matches with previous basketball-related research [29,34,35] and is also consistent with the results of decision-making assessments applied in other youth team sports [56]. However, response time could not differentiate the performance levels. The comparison of these results with those of other studies is limited because depending on the instruction (i.e., answer as fast as possible vs. answer as precise as possible) different response times must be expected [57]. The findings regarding response time can be explained by focusing on expert advantages in cognitive factors connected to decision-making skills. For example, elite athletes can anticipate how a game situation will evolve based on their contextual knowledge on probabilities that a distinct event will occur [22]. Thus, the youth national team players in the current study may not have decided on an earlier option because they anticipated an even better option. This is supported by the descriptive statistics that indicate slower response times in elite youth athletes (see Table 1). Further, the smaller variability in results of the elite athletes suggests that they were answering in an equal time frame. In contrast, the non-elite youth athletes were not able to hit this time frame consistently, rather they were answering too early. From a practical point of view, such behavior is effective because basketball players are eager to use as much information as possible to make accurate decisions while also trying to execute their decisions just at the right moment to make a successful pass. Another explanation may be differences in personality traits of all youth athletes apart from their basketball-specific expertise. Previous research has shown that the predisposition to state or action orientation can influence the decision-making skills of basketball players to such an extent that action-oriented players were faster in decision-making [58]. Athletes with such a predisposition can most likely be found in both groups and thus performance levels could not be differentiated regarding response time.

With respect to the second objective, the results support the external validity of the assessment. The diagnostic results correlate with assists and turnovers per game but not with the assist-turnover ratio (H2). The response time contributed to the explanation of both significant models which indicates that players who react faster in the assessment produce more assists and more turnovers in a game (see Figure 4). This study is among the first to investigate the relationship between elite athletes’ results in a decision-making assessment and their performance data. Thus, the findings cannot be compared to those of other studies. The ambivalence in the findings (i.e., faster response times in the assessment are related to more assists as well as more turnovers) can be explicated in a practical context: During a basketball game, a player needs to make a quick decision to execute, for example, a shot, dribbling or a pass. If the player decides too early, he might turn the ball over. Therefore, the correlation between response time and assists as well as turnovers per game is reasonable. However, response accuracy did not further contribute to the explanation of the performance data. This might be due to the moderate difficulty of the task which could have led to a ceiling effect within the elite athletes. This assumption is highlighted by the high examined level of response accuracy in the elite athletes (i.e., 83.99 ± 9.19%; see Table 1). The fact that the diagnostic results do not correlate with the assist-turnover ratio may be due to the investigated sample that comprised all playing positions. With respect to tactical considerations, each player is assigned certain responsibilities depending on his individual strengths particularly within competition [3]. However, it should be considered that roles in youth basketball teams are usually less distinguished compared to adult teams due to educational guidelines [59]. This is illustrated for example by the distribution of assists in the sample of the current study (i.e., elite youth basketball players from the German U16 national team): Seven players share approximately 85% of the assists in a range between 1.25 and 2.67 assists per game. As many teams play with a seven or eight-player rotation [60], passing is a crucial skill for all players of this youth team. Nevertheless, outside players (i.e., point guard, shooting guard, and small forward) may be more involved in passing situations than inside players (i.e., power forward and center). This is highlighted, comparing the performance data of outside and inside players in the 15 international games via t-test for independent variables. The supplemental analysis revealed significant differences between these positional groups concerning assists (*t*(11) = 3.44, *p* < 0.01) and the assist-turnover ratio (*t*(11) = 3.63, *p* < 0.01) (see Supplementary Materials, Table S2). Sampaio et al. [61] confirmed the discriminatory power of assists comparing adult guard, forward, and center players while acknowledging the importance of passing skills particularly for guards. However, the research investigating the performance data of elite basketball players according to playing positions is sparse. Another explanation addresses the peculiarities of game pace in youth basketball. Previous research demonstrated that elite youth basketball teams played less static and exhibit a higher frequency of fastbreaks compared to professional adult basketball teams [62]. With higher game pace, inaccuracies (e.g., turnovers) increase [4] which may affect the assist-turnover-ratio in the present study. Moreover, in the process of recording the performance data, not all passing decisions are registered because not every pass leads to an assist or turnover. Correct passing decisions can also lead to a drawn foul, a missed shot or another pass which is followed by an assist, a turnover, a drawn foul, or a missed shot. However, such information is not included in the official FIBA box scores. To adjust the performance data accordingly a retrospective video-based evaluation of the analyzed games would have been necessary, which was not possible within the scope of this study.

This was one of the first studies to use performance data for external validation of a decision-making assessment in youth basketball. Thus, methodological challenges associated with both diagnostic instruments and sports data have been addressed by embracing sufficient control and external validity in the development of the assessment [43]. In other studies, the related concept of ecological validity has been evaluated by using questionnaires to determine participants’ opinions on how closely the video scenes replicate the on-court reality [63]. Such game-likeness ratings offer relevant information about the motivational aspects of players completing a cognitive assessment. However, they are not contributing to the question of whether the assessments’ measures are related to game performance. Therefore, the presented approach is promising for external validation of such diagnostic instruments in team sports. The sparse basketball-related research concerning the linkage between cognitive performance factors and performance data supports the association of fundamental cognitive performance parameters with performance data also investigated in the current study [44,49]. Thus, the findings of this study underline the importance of cognitive factors for basketball players’ performance and contribute to a better understanding of this complex construct.

The current study expands the state of research by developing an assessment that uses stimuli from a first-person perspective. Former research primarily incorporated stimuli from a third-person perspective which lacked representation of the on-court reality. Within the current study, this problem is addressed and thus higher validity of measures and data can be expected [21]. A further strength of this study is the participating youth athletes. On the one hand, basketball players of the German U16 national team were tested who are also targeted in a practical context applying such assessments. The high selection level of the elite athletes resulted in a rather small and homogenous sample. Differences within such preselected groups are difficult to prove due to limited variance [64]. Indeed, this highlights the value of the results gathered. On the other hand, athletes of the same age group with experience in other sporting domains and considerable fitness levels were examined. In previous studies, control groups differed in age and did not show a comprehensive background in sports [65]. The influence of age and confounding variables connected to general expertise in sports (e.g., aerobic endurance) is therefore diminished by the design of the groups. Detected differences among the performance levels can thus most likely be explained by basketball-specific expertise. However, in addition to the strengths outlined, limitations should also be considered when interpreting the results of this study. First, the overall sample size was small due to the sampling criteria which is a shared issue of previous research evaluating decision-making assessments in basketball [29,34,35] and many youth team sports [56]. Second, within this study, the proper passing options were determined a priori based on the expert evaluation. However, the successful execution of a made decision depends on a player’s technical skills [8] and is also influenced by contextual factors (e.g., the status of time or score) [66] which is usually not represented within laboratory settings. Third, a common limitation of video-based assessments without a specific response is the lack of perception-action-coupling. Mann et al. [67] found that simplified movements potentially fail to reflect perceptual-motor expertise and that the implementation of a specific response would increase the performance of the participants. Even though the answer was conducted orally after pressing a buzzer in this study, significant discrimination among the performance levels was detected. This emphasizes the validity of the decision-making assessment.

For the further development of the assessment, more difficult video stimuli may be implemented to detect differences between athletes at the highest performance level regarding response accuracy. Additionally, sport-specific questionnaires may be applied complementary to the assessment of decision-making skills to control for athletes’ predisposition to action or state orientation (e.g., Action Control Scale Sport, ACS-Sport) [68]. Such an additional investigation might be instrumental to enable the demonstration of differences on both performance levels with respect to response time. Furthermore, the implementation of a specific response (e.g., passing a real basketball) and linked recording of data (e.g., via ball-implemented technology) should be considered in future studies. Increasing the task specificity can contribute to the assessments’ sensitivity. On the one hand, it can be expected that the differences between the performance levels will become even stronger. On the other hand, players from different playing positions can be expected to perform better in situations that they proficiently solve in a game. Such an enhancement has been shown to be beneficial for the assessment of the decision-making skills of elite youth soccer players [69]. Besides, options like shooting or driving towards the basket should be incorporated as possible options besides passing the ball. However, it is challenging to reproduce the full complexity of the game using video stimuli while maintaining a reasonable assessment scope. Therefore, the use of a graduated scoring system with the best option scoring higher than the least good option can contribute to the improvement of the assessment [37]. In addition, the adjustment of the performance data should be focused: On the one hand, all correct passing decisions should be considered even if they do not lead to an immediate score. On the other hand, the data of a whole season should be analyzed to get more insights into the linkage between the diagnostic results and performance data. In the current study, all games of the German U16 national team were analyzed (i.e., 15 international games in preparation and competition). However, not every player was on the roster in every game due to reasons of selection in the preparation period, management of the physical load, or decisions of the coaching staff during competition. In-season games in national youth competitions are usually played weekly allowing all players to be part of the roster on regular basis. Further, real game performance data were analyzed in the current study to examine the assessments’ external validity which is inherently influenced by playing time. However, if performance data are utilized for different purposes in future studies, the influence of playing time should be acknowledged and data adjustments (e.g., analyzing assists or turnovers per 100 min played) [44,49] should be taken into consideration. Besides, advanced statistics may be analyzed instead of the distinct variables (i.e., assists and turnovers per game) to externally validate enhanced assessments. For example, the offensive adjusted plus-minus may be convenient which represents a player’s marginal offensive contribution compared to an average player [70]. The application of such measures enables deeper insights into the relationship between the diagnostic results and the offensive impact of a basketball player on his team’s success. However, such an analysis is only conducive if the assessment also resembles the full complexity of offensive decision-making.

Future studies should also address the technological development that by now has established reliable methods to assess cognitive performance measures [71]. In this regard, the in situ assessment should be contemplated which is a promising approach in basketball [72]. Even more interesting is the investigation of decision-making skills in virtual reality environments where 360° video stimuli are presented through head-mounted displays. In contrast to in situ settings, virtual reality environments advance, for example, regarding greater control or the reproducibility of relevant game situations [71]. Research supports the beneficial effects of this technology for both the assessment and training of decision-making skills in team sports [56,73]. However, participants in the current study were not required to turn their heads to collect relevant information to prepare their decisions. Although it seems imperative to consider this technology to investigate, for example, the offensive decision-making skills of center players in basketball who typically attack with the back to the basket. Moreover, no defender was impairing the vision of the participants in the current study as the participants would not have been able to overcome vision impairment by changing their point of view during the assessment. However, research shows that an approaching defender can lead to a change in movement execution and gaze behavior during a shooting task in basketball [74,75,76]. Within both the in situ and the virtual reality setting, such investigations are possible. Overall, it should be noted that coaches and clubs widely do not have access to cognitive laboratories equipped with the necessary advanced technology for in situ investigations. Thus, virtual reality might also be the tool of choice for advanced testing in the process of talent identification because of the affordable price for head-mounted displays and 360° camera systems [73]. As an alternative to video-based assessments, various tools have been established to systematically observe decision-making in passing situations and other play actions in basketball (e.g., BALPAI) [77]. However, the application of such tools in a practical context is challenging due to the large number of items to be observed in real-time. Moreover, the observers must be trained in advance to ensure high inter and intra-observer reliability. Another alternative approach is offered by naturalistic decision-making research. In contrast to laboratory settings, studies with such an approach assess decision-making more comprehensively and take the contextual constraints into account [78]. Considering the complexity of the game of basketball, exploring decision-making skills in a realistic context can yield promising insights into athletes’ decision-making.

## 5. Conclusions

The assessment is applicable to investigate decision-making skills in youth basketball. It allows the discrimination of basketball players from other youth athletes focusing on response accuracy. Moreover, the discrimination of elite youth basketball players is possible with respect to response time. The assessment can be used as an additional tool in the process of talent identification to inform coaches regarding players’ decision-making skills. Due to the multifactorial character of sports performance, it should only be applied in combination with other established testing instruments to execute a holistic and sustainable talent diagnosis.

## Figures and Tables

**Table 1:** Descriptive statistics for the diagnostic results separated by performance level serving as predictor variables in the logistic regression analysis.

Variables	Elite ^a^	Non-Elite ^b^
	(*n* = 17)	(*n* = 17)
	*M* ± *SD*	
Response accuracy (%)	83.99 ± 9.19	57.35 ± 18.16
Response time ^c^	0.00 ± 0.31	−0.35 ± 0.92

^a^ Elite = Youth basketball players of the German U16 national team. ^b^ Non-elite = Students of a sports class. ^c^ This variable was z-standardized in reference to the elite athletes and is as such negatively coded, i.e., a lower value represents a higher performance.

**Table 2:** Results of the logistic regression analysis for the determination of the elite performance level according to the diagnostic results.

Variables	Logistic Regression Coefficients				Omnibus-Tests		
	*b*	Wald	*p*	*e^b^* [95% CI]	χ^2^ (*df*)	*p*	Nagelkerke’s *R*^2^
Constant	−8.99						
Response accuracy (%)	0.12	8.24	<0.01	1.13 [1.04; 1.23]	20.39 (2)	<0.001	0.60
Response time ^a^	−0.02	0.00	0.99	0.98 [0.15; 6.66]			

**Note.** *N* = 34. CI = confidence interval. ^a^ This variable was z-standardized in reference to the elite athletes and is as such negatively coded, i.e., a lower value represents a higher performance.

**Table 3:** Descriptive statistics for the diagnostic results and the performance data serving as predictor and external criterion variables in the multiple regression analysis.

Variables	*M* ± *SD*
**Decision-Making Assessment**	
Response accuracy (%)	82.91 ± 9.55
Response time ^a^	−0.03 ± 0.25
**Performance Data**	
Assists per game	1.24 ± 0.91
Turnovers per game	1.64 ± 0.79
Assist-Turnover ratio	0.73 ± 0.49

**Note.** Only players were evaluated who competed in at least 20% of the games (*n* = 13). ^a^ This variable was z-standardized in reference to the elite athletes and is as such negatively coded, i.e., a lower value represents a higher performance.

**Table 4:** Results of the multiple regression analysis for the determination of the relationship between the diagnostic results and the performance data.

Variables	Model 1			Model 2			Model 3		
	Assists per Game			Turnovers per Game			Assist−Turnover Ratio		
	*B*	ß	*SE*	*B*	ß	*SE*	*B*	ß	*SE*
Constant	0.63		1.87	1.98		1.60	0.04		1.20
Response accuracy (%)	0.01	0.07	0.02	−0.01	−0.06	0.02	0.01	0.15	0.01
Response time ^a^	−2.44 *	−0.68	0.85	−2.30 *	−0.73	0.73	−0.91	−0.47	0.55
									
*R* ^2^	0.49			0.51			0.29		
*F* (2,10)	4.82 *			5.23 *			2.01		

**Note.** * *p* < 0.05. Only players were evaluated who competed in at least 20% of the games (*n* = 13). ^a^ This variable was z-standardized in reference to the elite athletes and is as such negatively coded, i.e., a lower value represents a higher performance.

## Data Availability

The raw data supporting the findings of this study will be made available by the corresponding author upon reasonable request.

## Supplementary Materials

The following are available online at https://www.mdpi.com/1660-4601/18/5/2331/s1, Table S1: Box scores of the international games, Table S2: Descriptive statistics for the performance data separated by positional groups.
