# Supplementary material for: Decision-Making Skills in Youth Basketball Players: Diagnostic and External Validation of a Video-Based Assessment
Source: Int J Environ Res Public Health. 2021 Feb 27;18(5):2331. doi: 10.3390/ijerph18052331 (PMC7967709; doi:10.3390/ijerph18052331)
Supplement: Supplementary file 1 [file ijerph-18-02331-s001.pdf]

Supplementary Materials

Table S1. Box scores of the international games.

No.	Date	Fixture	Data availability
1	June 26, 2019	Germany vs. France	The box score of this game was not prepared for online publication. The first author of the manuscript archived the box score after the game and will make it available upon reasonable request.
2	June 28, 2019	Germany vs. France	The box score of this game was not prepared for online publication. The first author of the manuscript archived the box score after the game and will make it available upon reasonable request.
3	July 18, 2019	Germany vs. Poland	http://www.fibalivestats.com/u/DBB/1289328/
4	July 19, 2019	Germany vs. Poland	http://www.fibalivestats.com/u/DBB/1289329/
5	July 31, 2019	Lithuania vs. Germany	http://www.fibalivestats.com/u/LBS/1309410/
6	August 1, 2019	Estonia vs. Germany	http://www.fibalivestats.com/u/LBS/1309411/
7	August 2, 2019	Latvia vs. Germany	http://www.fibalivestats.com/u/LBS/1147654/
8	August 6, 2019	Germany vs. Serbia	http://www.fibalivestats.com/u/DBB/1289330/
9	August 9, 2019	Italy vs. Germany	http://www.fiba.basketball/europe/u16/2019/game/0908/Italy-Germany#|tab=boxscore
10	August 10, 2019	Germany vs. Russia	http://www.fiba.basketball/europe/u16/2019/game/1008/Germany-Russia#tab=boxscore
11	August 11, 2019	Germany vs. Croatia	http://www.fiba.basketball/europe/u16/2019/game/1108/Germany-Croatia#|tab=boxscore
12	August 13, 2019	Spain vs. Germany	http://www.fiba.basketball/europe/u16/2019/game/1308/Spain-Germany#|tab=boxscore
13	August 14, 2019	Germany vs. Slovenia	http://www.fiba.basketball/europe/u16/2019/game/1408/Germany-Slovenia/#tab=boxscore
14	August 16, 2019	Bosnia and Herzegovina vs. Germany	http://www.fiba.basketball/europe/u16/2019/game/1608/Bosnia-and-Herzegovina-Germany/#tab=boxscore
15	August 17, 2019	Latvia vs. Germany	http://www.fiba.basketball/europe/u16/2019/game/1708/Latvia-Germany#|tab=boxscore

Note. Games 9-15 took place at the 2019 FIBA U16 European Championship while Games 1–8 were played in preparation for this tournament.

Table S2. Descriptive statistics for the performance data separated by positional groups.

**Variables**	**Outside** [a] **(*n* = 7)**	**Inside** [b] **(*n* = 6)**
	M ± *SD*	
Assists per game	1.82 ± 0.70	0.56 ± 0.60
Turnovers per game	1.87 ± 0.67	1.38 ± 0.90
Assist-Turnover	1.05 ± 0.33	0.35 ± 0.37

[a] Outside = point guard, shooting guard and small forward. [b] Inside = power forward and center.
